# Impact of *Bacillus* in fermented soybean foods on human health

**DOI:** 10.1186/s13213-021-01641-9

**Published:** 2021-07-17

**Authors:** Trishala Gopikrishna, Harini Keerthana Suresh Kumar, Kumar Perumal, Elavarashi Elangovan

**Affiliations:** Department of Biotechnology, Sri Ramachandra Faculty of Biomedical Sciences & Technology, Sri Ramachandra Institute of Higher Education and Research (SRIHER), Deemed to be University, Chennai, India

**Keywords:** Fermented soybean foods (FSF), *Bacillus* spp., Bioactive compounds, Health risks, Food safety

## Abstract

**Purpose:**

Fermented soybean foods (FSF) is popularly consumed in the South-East Asian countries. *Bacillus* species, a predominant microorganism present in these foods, have demonstrated beneficial and deleterious impacts on human health. These microorganisms produce bioactive compounds during fermentation that have beneficial impacts in improving human health. However, the health risks associated with FSF, food pathogens, biogenic amines (BAs) production, and late-onset anaphylaxis, remain a concern. The purpose of this review is to present an in-depth analysis of positive and negative impacts as a result of consumption of FSF along with the measures to alleviate health risks for human consumption.

**Methods:**

This review was composed by scrutinizing contemporary literature of peer-reviewed publications related to *Bacillus* and FSF. Based on the results from academic journals, this review paper was categorized into FSF, role of *Bacillus* species in these foods, process of fermentation, beneficial, and adverse influence of these foods along with methods to improve food safety. Special emphasis was given to the potential benefits of bioactive compounds released during fermentation of soybean by *Bacillus* species.

**Results:**

The nutritional and functional properties of FSF are well-appreciated, due to the release of peptides and mucilage, which have shown health benefits: in managing cardiac disease, gastric disease, cancer, allergies, hepatic disease, obesity, immune disorders, and especially microbial infections due to the presence of probiotic property, which is a potential alternative to antibiotics. Efficient interventions were established to mitigate pitfalls like the techniques to reduce BAs and food pathogens and by using a defined starter culture to improve the safety and quality of these foods.

**Conclusion:**

Despite some of the detrimental effects produced by these foods, potential health benefits have been observed. Therefore, soybean foods fermented by *Bacillus* can be a promising food by integrating effective measures for maintaining safety and quality for human consumption. Further, in vivo analysis on the activity and dietary interventions of bioactive compounds among animal models and human volunteers are yet to be achieved which is essential to commercialize them for safe consumption by humans, especially immunocompromised patients.

## Introduction

Fermented foods are defined as the food produced through the activity of microorganisms and the enzymatic conversion of food components with better shelf-life, safety, nutritional, and therapeutic properties. These foods comprise of bioactive molecules, vitamins, and other necessary contents with enhanced availability due to the process of fermentation (Rezac et al. 2018). Fermented foods are prepared by the action of microorganisms (natural or starter culture), under the appropriate environmental conditions like temperature, pH, and moisture content. The live microorganisms present in the fermented foods help improving gut microbiota, which in turn improves the gastrointestinal health and aids to overcome health problems like diabetes and cardiovascular diseases (Melini et al. 2019).

There are several varieties of fermented foods using different substrates like milk, cereals, vegetables, legumes, root crop, meat, and fish that are prepared in their own way. A brief description of the diverse variety of fermented soybean products made from soybean is discussed in this review article. The preparation of fermented soybean foods (FSF) utilizes substrates like soybean, black gram, and locust beans. The major FSF are soy paste, soy sauce, tempeh, natto, sufu, soy nuggets, and soy yogurt, which are traditionally as well as industrially prepared in Asian countries like Korea and Japan. The *Bacillus* spp. present in the FSF includes *B. amyloliquefaciens*, *B. cereus*, *B. circulans*, *B. licheniformis*, *B. sphaericus*, *B. subtilis*, and *B. thuringiensis.* However, *B. subtilis* is the dominant functional bacterium in the FSF. Lactic acid bacteria (LAB), filamentous fungi, yeasts, *Aspergillus*, *Torulopsis*, *Zygosaccharomyces*, and *Rhizopus* are some of the other microorganisms, which are identified in the FSF (Frias et al. 2017).

The current review aims to summarize the beneficial and harmful impacts of *Bacillus* spp., in the fermented soybean food (FSF). Several studies have proved that the FSF, which are rich in protein, have numerous applications. However, there is no systematic review focusing on the positive impacts of these microorganisms in the FSF opposed to its baneful impacts. This review article aids the researchers, who are in need of the information regarding the importance of *Bacillus* species on health, which are present in FSF. This article also highlights the side effects on consumption of these FSF and also pinpoints the possible ways to produce safe foods. Hence, the in-depth analysis of the various research findings on fermented soy products may help researchers understand its beneficial and harmful impacts on human health.

### *Bacillus* in fermented soybean foods

#### Ingredients involved in preparing FSF

**Fig. 1:**
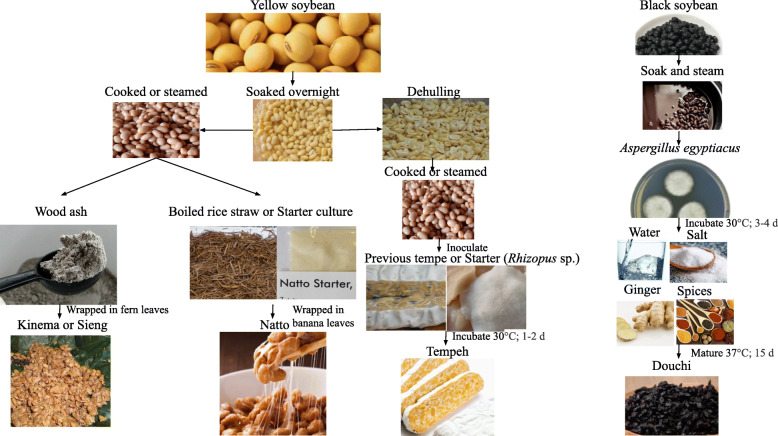
Fermentation process of yellow soybean and black soybean

Fermentation of soybean requires soaking and boiling of the soybean, followed by splitting the cotyledons to speed up the process of fermentation by the *Bacillus* spp. In order to maintain an alkaline condition during fermentation, about 1% of firewood ash can be added, and to maintain an ambient temperature (20–35 °C), the soybean grit is wrapped in the leaves of banana, bamboo, or ginger. *Calliparpa aroria or Phrynium* spp. is used for processing foods like natto, aakhone, bekang, axone, and peruyyan. Bamboo baskets or jute bags lined with either banana leaves or fern leaves are used for storing and processing foods like tungrymbai, hawaijar, and axone (Singh et al. 2014a, 2014b). After 1–2 days, fermentation is indicated by the presence of white viscous layer and slight ammonia odor. This process of fermentation helps in elevating the shelf life to 2–3 days, and the same can be preserved even for a month by drying it in the sun for about 2–3 days (Tamang 2015). The fermentation process for yellow soybean and black soybean to produce kinema or sieng, natto, tempeh, and douchi respectively is depicted (Fig. 1).

**Fig. 2:**
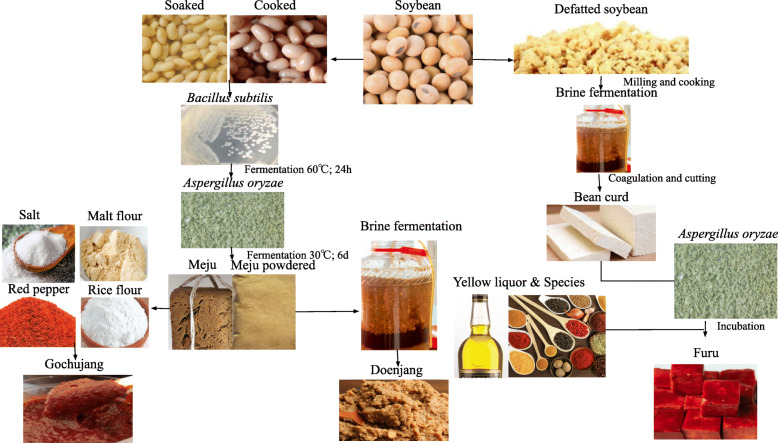
Fermentation process of cooked and defatted soybean

The fermentation process involved in making gochujang, doenjang, and furu from cooked and defatted soybean is illustrated (Fig. 2).

**Fig. 3:**
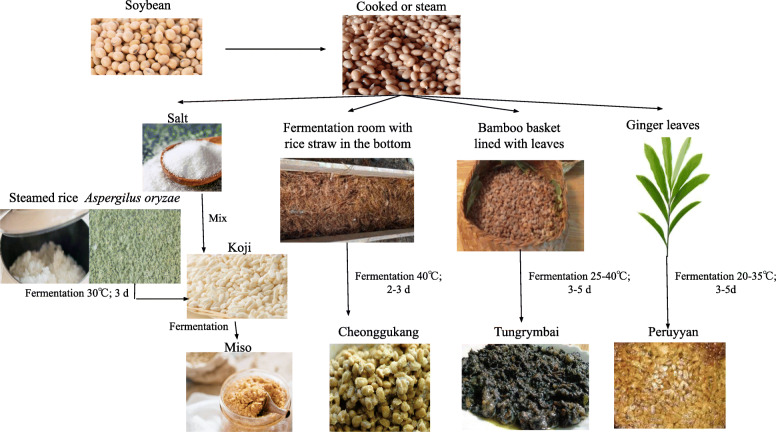
Fermentation process of cooked or steamed soybean

The process involved in fermenting soybean to produce miso, cheonggukjang, tungrymbai, and peruyyan is portrayed (Fig. 3).

**Fig. 4:**
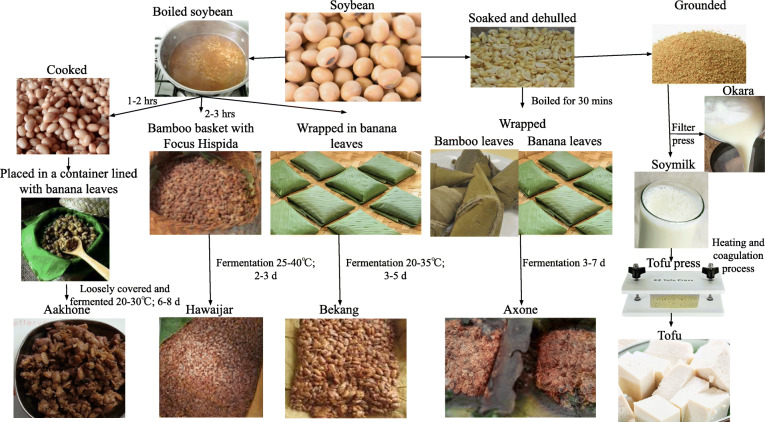
Fermentation process of boiled and dehulled soybean

The fermentation of soybean to produce aakhone, hawaijar, bekang from the boiled soybean and axone, and tofu from dehulled soybean is represented (Fig. 4).

#### Dominant microorganisms present in FSF

LAB and *Bacillus* species are widely present in many fermented foods and beverages. *Bacillus* spp. (*Bacillus circulans*, *B. licheniformis*, *B. sphaericus*, *B. subtilis*, and *B. thuringiensis*) are the dominant bacteria in the FSF, which help in the process of fermentation. There are other microorganisms present in FSF like *Aspergillus sp.*, *Rhizopus*, *Mucor hiemalis or Actinomucor elegans*, LAB like *Lactobacillus bulgaricus*, *Streptococcus thermophilus*, *Leuconostoc*, *and Pediococcus*. The FSF products are commonly consumed by the Southeast Asian countries (Dimidi et al. 2019). The geographical distribution of the common FSF of the Southeast Asian countries is depicted in Table 1. Some of the uncommon FSF from which *Bacillus* spp. are isolated include bhallae, bikalga, ce-lew, kanjang, meitauza, pepok, sieng, tamari shoyu, and thua nao (Tamang et al. 2015).

**Table 1:** Geographical distribution of common fermented soybean food products of Southeast Asian countries

Country	Fermented soybean food products	Functional microorganisms which aid in fermentation	Other than *Bacillus* spp.	Reference
Japan	Natto	*Bacillus subtilis*	*Aspergillus oryzae*	Liu 2012)
Japan	Miso	*Bacillus subtilis*	*Pediococcus acidilactici*, *Leuconostoc mesenteroides*, *Micrococcus halobius*, *Aspergillus oryzae*, *Zygosaccharomyces rouxii and Torulopsis sp*	Tamang et al. (2016)
China	Furu	*B. pumilus*, *B. megaterium*, *B. stearothermophilus*, *B. firmus*	*Staphylococcus hominis*	Lin et al. (2016)
Korea	Cheonggukjang	*Bacillus amyloliquefaciens*, *B. licheniformis*, *Bacillus megaterium*, *and B.subtilis*	*Leuconostoc*, *Lactobacillus*, *Pseudomonas*, *Pantoea and Weissella genera*	Hong et al. (2012)
Korea	Gochujang	*B. velegensis*, *B. amyloliquefacious*, *B. subtilis*, *B. licheniformis*, *Oceanobacillus* spp.	*Zygosaccharomyces*, *Candida lactis*, *Zygorouxii*, *Aspergillus*, *Penicillium*, *Rhizopus*	Nam et al. (2012)
Mizoram state of India	Bekang	*B. subtilis*, *B. licheniformis*, *B. pumilus*, *Bacillus cereus*, *B.amyloliquefaciens*	*Debaryomyces hansenii*, *Pichia burtonii*	Chettri and Tamang (2014); Singh et al. (2014a, 2014b)
Meghalaya state of India	Tungrymbai	*B. subtilis*, *B. pumilus*, *B. licheniformis*, *B. amyloliquefaciens*, *Lactobacillus brevis*,	*Vagococcus carniphilus*, *Debaryomyces hansenii*, *Pic. burtonii*,	Chettri and Tamang (2015); Singh et al. (2014a, 2014b)
Nepal, Bhutan, India	Kinema	*B. subtilis*, *B. licheniformis*, *B. cereus*, *B. circulans*, *B. thuringiensis*, *B. sphaericus*	*Geotrichum candidum.*	Chettri et al. (2016); Chettri and Tamang (2015); Singh et al. (2014a, 2014b)
Manipur state of India	Hawaijar	*B. subtilis*, *B. licheniformis*, *B. amyloliquefaciens*, *B. cereus*,	*Alcaligenes sp.*, *Providencia rettgers*	Singh et al. (2014a, 2014b)
Korea	Doenjang	*B. subtilis*, *B. licheniformis*, *B. pumilus*,	*Mucor plumbeus*, *Aspergillus oryzae*, *Debaryomyces hansenii*, *Leuconostoc mesenteroides*, *Tor. halophilus*, *Lactobacillus sp.*	Chang et al. (2013); Kim et al. (2016a)
China, Taiwan	Douchi	*B. amyloliquefaciens*, *B. subtilis*,	*Aspergillus oryzae*	Yang et al. (2019a, 2019b)
Indonesia (Origin), Netherlands, Japan	Tempeh	*Bacillus pumilus and B.brevis*	*Rhizopus spp.*, *Lactobacillus casei*	Frias et al. (2017)
Arunachal Pradesh state of India	Peruyaan	*B.subtilis*, *B. amyloliquefaciens*,	*Vagococcus lutrae*, *Pediococcus acidilactici*	Singh et al. (2014a, 2014b)
Nagaland state of India	Axone Aakhone	*Bacillus subtilis*	*–*	Deb and Jamir (2018)
Cambodia, Laos	Sieng	*Bacillus subtilis*	*–*	Sopheap et al. (2019)
Korea	Meju	*Bacillus cereus*, *B. circulans*, *B. licheniformis*, *B. megaterium*, *B. mesentricus*, *B. subtilis*, *B. pumilus*	*Aspergillus spp*, *Botrytis cinerea Rhizopus oryzae*, *Rhodotorula flava*, *Zygosaccharomyces japonicus*, *Lactobacillus sp*, *Ped. acidilactici*	Jang et al. (2019)

#### *Bacillus* species and its enzymes

The proteins present in the soybean are heat stable, and they can be fermented without degradation of their chemical composition. The soybean fermented using *Bacillus* spp. occurs in an alkaline condition. (Sanjukta and Rai 2016; Seo and Lee 2004). The *Bacillus* spp*.* present in the fermented foods hydrolyze the substrate and produce enzymes such as nattokinase, phytase, amylase, protease, cellulase, and lipase. However, the fermentation conditions need to be consistent and optimal for the growth of *Bacillus* species to produce such enzymes (Nguyen and Nguyen 2020). These enzymes help to break down the complex compounds to simple biomolecules. For example, the starch present in the soybean is converted into sugar by the enzyme amylase. Likewise, protease is used in the conversion of proteins to amino acids (Rai et al. 2017). *Bacillus subtilis* which is widely existing in the natural environments produces protease and esterase that degrade proteins and fats in the soybean. *Bacillus* spp., like *Bacillus subtilis* producing proteolytic enzymes, hydrolyze soybean proteins (glycinin and β-conglycinin), which leads to the production of specific bioactive peptides (Sanjukta and Rai 2016). These peptides are inactive within the parent protein, and they are released only upon enzymatic hydrolysis during fermentation and gastrointestinal digestion (Tamang et al. 2016). According to a study, *Bacillus siamensis* present in the FSF improves the nutritional properties by reducing β-sheet structure by 43.2% and destroying the original structure of soybean protein which effectively promotes protein digestibility (Zheng et al. 2017).

#### Fermentation using starter cultures

Biomolecules aid in biological activities like fermentation. The enzymes catalyze several chemical and enzymatic reactions that lead to the production of a characteristic sticky material called mucilage, which is a mixture of poly gamma glutamic acid (PGA) and fructan produced by *Bacillus subtilis* var. natto. The mucilage produced from this type of fermentation has shown to have therapeutic potential against various chronic diseases. Hence, the fermented soybean can be utilized as a functional food material with potential applications in food, cosmetics, and medicines and also in the formation of characteristic aroma, flavor, and various compositional changes (Chettri and Tamang 2014). Since *Bacillus* spp., present in the fermented soybean, has potential benefits, these microorganisms are used as a starter culture for effective fermentation.

*B. subtilis* subsp. *subtilis* BEST195 is the starter strain used for the production of FSF (natto). *B. subtilis* strains are spore-forming bacteria. These are commonly found in dried rice straw which could be used to initiate natto fermentation (Kamada et al. 2015). *Bacillus siamensis* (D2-2) and *Bacillus subtilis* (D12-5) have been reported to serve as starter cultures for fermenting low-salt (6.5–7.5% NaCl) doenjang, which showed both proteolytic and anti-pathogenic activities (Jeon et al. 2016). Studies on the industrially prepared flavor-rich doenjang are reported to have similarities to the traditional doenjang which have been found to mimic the fermentation of autochthonous mixed starters like *A. oryzae* MJS14, *B. subtilis* D119C, and *Tetragenococcus halophilus* 7BDE22. The pilot scale manufactured doenjang showed highest consumer acceptability, a rich flavor, and assured safety, and it was produced within 5 weeks (Lee et al. 2018a, 2018b). The FSF such as natto, miso, tauco, gochujang, douchi, cheonggukjang, doenjang, tofu, tempeh, meju, and bean curd are commercially available in retail markets across the East and Southeast Asian countries like Japan, China, Indonesia, and Korea. Some soybean foods like tofu are also available in countries like the USA and India (Chen et al. 2012; Vann et al. 2020). These commercially available fermented foods are fermented by using starter cultures such as *Bacillus subtilis* for preparing kinema, tungrymbai, bekang, and natto (Tamang 2015). *Aspergillus oryzae* are used for producing furu (Lin et al. 2016) gochujang, doenjang (Shin and Jeong 2015), and miso (Nout 2015). *Rhizopus* species and *Aspergillus egyptiacus* can be used to produce tempeh and douche respectively (Nout 2015).

### Health benefits

Soybean fermentation releases small peptides and mucilage, which have shown to be a potential source for numerous health benefits (Chatterjee et al. 2018). The microorganisms which aid the fermentation of soybean produces bioactive compounds and these compounds have beneficial effects. The bioactive compounds produced by the *Bacillus* species and its health benefits are illustrated in Table 2.

**Table 2:** Bioactive compounds produced by the *Bacillus* species and its health benefits

Microorganisms	Bioactive compounds	Health benefits	References
*Bacillus* species	Poly-γ-glutamic acid	Suppression in the elevation of post prandial blood glucose level	Chettri et al. (2016)
*Bacillus* species	Isoflavone aglycone	Flavonoid bioconversion	Kim et al. (2018)
*Bacillus* species LM7	Bacillomycin D and surfactin	Antimicrobial activity	Lee et al. (2016a, 2016b)
*Bacillus subtilis* var. natto	Nattokinase and serine protease subtilisin	Fibrinolytic activity and tissue plasminogen activator can be increased	Mohanasrinivasan et al. (2017)
*Bacillus subtilis* 1423	Protease	Breaks down the complex protein to simpler molecules	Nguyen and Nguyen (2020)
*Bacillus subtilis* DC27	Serine protease	Fibrinolytic activity	Hu et al. (2019)
*Bacillus subtilis* natto O9516	ACE inhibitory peptides	Anti-hypertensive activity	Ibe et al. (2009)
*Bacillus subtilis*	Nattokinase	Anti-hyperlipidemic activity	Chen et al. (2018)
*Bacillus* natto TK-1	Lipopeptide	Biosurfactant activity	Cao et al. (2009)
*Bacillus subtilis*	Protease and esterase	Degradation of proteins and fats	Sanjukta and Rai (2016)
*Bacillus subtilis*	Proteolytic enzymes	Glycinin and β -conglycinin hydrolyzed for the production of bioactive peptides	Sanjukta and Rai (2016)
*Bacillus subtilis* var. natto	PGA and fructan (mucilage)	Antioxidant properties	Chettri and Tamang (2014)
*Bacillus subtilis* SN7	Bacteriocin	Biocontrol agent	Lee and Chang (2017)
*Bacillus subtilis* CSY 191	Surfactin	Inhibits growth of human breast cancer cells (MCF-7)	Lee et al. (2012)
*Bacillus subtilis* (natto)	Lipoproteins	Anti-inflammatory response	Rhayat et al. (2019)
*Bacillus subtilis* SHZ	Bioactive peptide and polyphenols	Antioxidant activity	Sanjukta and Rai (2016)
*Bacillus subtilis* N205 (BS205)	Isoflavone aglycones	Antioxidant properties	Ping et al. (2012)
*Bacillus subtilis*-SKm (BS-c)	α-amylase, and γ-GTP	Antioxidant property	Zheng et al. (2011)
*Bacillus subtilis* SC-8	Surfactin, fengycin and iturin.	Antifungal and antibiotic properties	Yeo et al. (2011a, 2011b)
*Bacillus subtilis* NT-6	Amphipathic peptide AMPNT-6	Antimicrobial effect	Xu et al. (2013)
*Bacillus subtilis* SCK-2	Peptide AMPC IC-1	Antimicrobial properties	Yeo et al. (2011a, 2011b)
*Bacillus amyloliquefaciens* FZB42	Fibrinolytic enzymes	Fibrinolytic activity	Huy et al. (2016)
*Bacillus amyloliquefacians* EMD 17	Surfactin	Antimicrobial activity	Lee et al. (2016a, 2016b)
*Bacillus amyloliquefacians*	Daidzein	Anti-diabetic property	Jeong et al. (2020)
*Bacillus amyloliquefacians*	1-Deoxynojirimycin	Alpha-glucosidase inhibitor	Cai et al. (2017)
*Bacillus amyloliquefaciens* SWJS22	β-glucosidase and protease	Antioxidant activity	Yang et al. (2019a, 2019b)
*Bacillus amyloliquefaciens* RWL-1	Isoflavones and phenolic contents	Antioxidant properties	Shahzad et al. (2020)
*Bacillus amyloliquefaciens*	Genistein	Neuroprotective effect	Khosravi and Razavi (2021)
*Bacillus licheniformis* B65-1	Phenylacetic acid	Inhibition of enteric pathogens	Kim et al. (2004)
*Bacillus licheniformis* 67	Poly-γ-glutamic acid	Anti-obesity effect	Choi et al. (2016)
*Bacillus licheniformis* (SFC)	Isoflavones aglycones	Anti-diabetic effect	Yang et al. (2014)

#### Fibrinolytic activity

Fibrin is a protein component, which is responsible for the clotting of blood at the wound site. However, excess accumulation of fibrin in the blood vessels can result in serious impairment to the blood circulation, which leads to thrombosis. In this view, FSF (natto) has been found to enhance fibrinolytic activity in plasma and in the production of tissue plasminogen activator. *Bacillus subtilis* var. *natto* produces the enzyme nattokinase and serine protease called subtilisin, which is proved to have fibrinolytic activity along with the increase in tissue plasminogen activator. The reduction in platelet aggregation in turn reduces the blood viscosity (Mohanasrinivasan et al. 2017).

Recent studies have shown that various potent fibrinolytic enzymes can be produced from different kinds of traditional FSF, for example Japanese natto, Chinese fermented paste, fermented red bean, douchi, doenjang, cheonggukjang, and Indonesian tempeh. The fibrinolytic activity of DFE27 enzyme, serine protease produced by *Bacillus subtilis* DC27 in douchi, has reported to show approximately 90% of fibrinolytic activity in the plasminogen-rich plate. This indicates that the purified DFE27 can directly hydrolyze fibrin and also convert plasminogen to plasmin, which makes this a potential fibrinolytic agent (Hu et al. 2019). Yet, another study proves that the traditional Vietnamese FSF, miso, which is fermented by *Bacillus amyloliquefaciens* FZB42, are capable of producing fibrinolytic enzymes ranging from 29.74 to 77.95 FU/g under a solid-state fermentation (Huy et al. 2016).

#### Prevention of hypertension

The biologically active peptides which are present in FSF like angiotensin I-converting enzyme (ACE)-inhibitory peptide exert an anti-hypertensive effect by *B. subtilis* natto O9516 (Vallabha and Tiku 2014). The ACE inhibitory peptides are generated by the proteolytic degradation of soybean protein fractions (glycinin and β-conglycinin) (Handa et al. 2020). A novel ACE-inhibitory peptide has been isolated from soybean meal which is fermented by *B. subtilis* natto that showed an activity of 84.1% with an IC50 value of 0.022 mg/ml (Wang et al. 2013). The results suggest that these ACE-inhibitory peptides obtained have a potential effect in the management of hypertension (Sanjukta and Rai 2016).

#### Protection against atherosclerosis

Cardiovascular diseases (CVDs) are the most leading cause of deaths, worldwide. Atherosclerosis is an arterial disease. It refers to the building up of plaque made up of fat, cholesterol, calcium, and other substances found in the blood. Over time, the hardening of plaque narrows the arteries, which limits the flow of oxygen-rich blood to the organs and other parts of the body. This leads to serious problems like heart attack, stroke, or even death (Gan et al. 2016). According to a study, polyamines present in shuidouchi may have the potential to increase longevity and reduce certain age-associated CVDs (Chen et al. 2019). Nattokinase (NK), which is an enzyme produced by *Bacillus subtilis* in the fermented soybean, possesses a strong anti-hyperlipidemic activity, which can prevent atherosclerosis. The study of Chen et al. reports natto to be effective in reducing cholesterol and its associated lipids. NK prevents atherosclerosis through its antioxidant property, which results in the reduction of lipid peroxidation and improved lipid metabolism (inhibition of low-density lipoprotein [LDL] oxidation) (Chen et al. 2018).

#### Protection against enteric pathogens in the intestine

Antimicrobial activity of the *Bacillus* species isolated from the various traditional FSF like cheonggukjang, doenjang, and meju that produces antimicrobial compounds like proteins, enzymes, lipopeptides, and bacteriocins. *Bacillus natto* TK-1 isolated from natto has been found to produce a lipopeptide that has a strong surface activity. These lipopeptides have found to show anti-adhesive effect against several bacterial pathogens such as *Botrytis cinerea*, *Fusarium moniliforme*, *Micrococcus luteus*, and *S. typhimurium* and food-borne pathogens like *Bacillus cereus* (Cao et al. 2009; Eom et al. 2014). Hence, these antimicrobial peptides could be used as an alternative to antibiotics for the treatment of bacterial/ fungal diseases as well as food preservatives (Lee et al. 2016a, 2016b).

An organic compound like phenylacetic acid, which is extracted from *Bacillus licheniformis* (B65-1), present in chungkookjang has shown to inhibit the growth of enteric pathogens like *Staphylococcus aureus* and *E. coli* (Kim et al. 2004). It is found that the lipopeptides are the major compounds responsible for the antibacterial and antifungal activities in the FSF. Yet, another study indicated that the production of surfactin by *B. amyloliquefaciens* EMD17 from cheonggukjang might be the responsible agent, which could be utilized as a starter strain for the fermentation of soybean foods (Lee et al. 2016a, 2016b). The bacteriocin produced by *B. subtilis* SN7 has potential to act as a biocontrol agent against *S. aureus and E. coli* O157:H7. Hence, this strain can be used as a starter culture in the preparation of cheonggukjang (Lee and Chang 2017).

#### Inhibitory response towards cancer

Cancer is an abnormal growth of cells that either localize at a particular site or metastasize throughout the body. Breast cancer accounts for 24.2% of new cancer cases and 15.0% of cancer deaths. Worldwide, the incidence and mortality of breast cancer ranks first in 154 and 104 countries, respectively (Cai and Liu 2019). Antitumor activity was observed in the biosurfactant present in natto, which is fermented using *B. natto* TK-1 (Sanjukta and Rai 2016). *B. subtilis* CSY191 isolated from doenjang produces surfactin, which inhibits the growth of human breast cancer cells (MCF-7) in a dose-dependent manner, with an IC50 value of 10 mg/ml at 24 h. The effect of *Bacillus* present in cheonggukjang was also studied on the growth of breast cancer MCF7 cells. It was found to downregulate the inflammation related genes and to also activate the transforming growth factor (TGF) pathway (Sanjukta and Rai 2016).

Shuidouchi has shown to contain polyamines like SPM (spermine) and SPD (spermidine). Human epidemiological studies reported that dietary polyamines are correlated in mitigating cancer and cardiovascular related mortality (Chen et al. 2019). A study confirmed that garlic-supplemented doenjang has been reported to demonstrate the enhancement of phenolics, flavonoids, and antioxidants along with improved anticancer activity against gastric and lung adenocarcinoma. This is due to the presence of high levels of free amino acids in garlic-supplemented doenjang, making it nutritionally valuable and also a source of functional foods (Bahuguna et al. 2019).

#### Immunostimulatory and anti-inflammatory activity

*B. subtilis* (natto) cells contain some of the active components, like peptidoglycans, lipoproteins, lipoteichoic acid, flagellin, and unmethylated CpG dinucleotides in bacterial DNA, which are known to bind to the toll-like receptors and induce cytokine response (Rhayat et al. 2019). Soybean fermentation using specific microorganisms have shown to reduce components responsible for immunoreactivity. Cheonggukjang is reported as a food with high immunostimulatory activity. This is due the presence of polysaccharides which increases the production of tumor necrosis factor alpha (TNFα). It is also reported that FSF such as doenjang and cheonggukjang exhibit anti-inflammatory action by modulating the expression of cyclooxygenase-2 (COX-2) and nitric oxide synthase (iNOS). TNF-α, Interleukin-6 (IL-6), and iNOS were used as markers for both immunostimulatory and inflammatory activity. Hence, the increase in TNF-*α*, IL-6, and iNOS might be interpreted as an increase in immunostimulatory activity by FSF (Choi et al. 2014).

#### Anti-obesogenic effect towards hyperlipidemia

Overweight and obesity are the risk factors for diabetes, hypertension, dyslipidemia, asthma, arthritis, and coronary heart disease (Otang-Mbeng et al. 2017). Anti-obesogenic effects have been reported in doenjang, which are fermented primarily by *Bacillus subtilis* and molds. Aglycones such as genistein and daidzein, which are produced by soybean during fermentation, are reported to increase hepatic carnitine palmitoyltransferase-1 (CPT-1) enzyme activity by upregulating CPT-1 transcription. This in turn results in increased expenditure of energy and ultimately leads to decrease in body fat and weight. Animal studies have also consistently proved that doenjang reduces hyperlipidemia in rats fed with a high fat/high cholesterol diet. It has been reported that prolonged intake of isoflavone-rich soybeans and FSF such as doenjang with a dosage of 40 g for 12 weeks seemed to decrease abdominal fat, which is important for preventing age-related chronic diseases (Cha et al. 2012; Woo et al. 2009).

#### Anti-allergic response

Food allergens like glycinin and β-conglycinin which are present approximately 30% in soybean meal are known to cause hypersensitivity in animals and also abnormalities like small bowel and diarrhea in newborn animals (nursery pigs) (Wang et al. 2014). According to a study, solid-state fermentation by microorganisms like *Bacillus subtilis*, *Lactobacillus casei*, and yeast could degrade these major allergens and reduce potential allergenicity of the soybean meal (SBM) (Yang et al. 2018).

#### Prevention of osteoporosis

Osteoporosis is a multifactorial disorder with low bone mass, intensified skeletal fragility, and impaired bone quality with a propensity to fracture (Rosen 2020; Sözen et al. 2017). According to recent statistics from the International Osteoporosis Foundation, worldwide, 1 in 3 women over the age of 50 years and 1 in 5 men are likely to experience osteoporotic fractures in their lifetime (Sözen et al. 2017). A study identified that cheonggukjang has increased the amount of isoflavone (aglycone) content. Its supplementation in SAMP6 mice could promote osteogenesis and inhibit osteoclastogenesis through the bone morphogenetic protein 2 (BMP2)/SMADs protein/osteoprotegerin (OPG) pathway. Hence, it is evidentially concluded that consumption of cheonggukjang is a potential complementary therapeutic food to prevent senile osteoporosis (Kim et al. 2019). According to a Japanese study, habitual intake of natto may be associated with a reduced risk of osteoporotic fractures in postmenopausal women (Kojima et al. 2019).

According to a meta-analysis for prospective studies of natto on bone mineral density (BMD), it is reported that natto has anti-osteoporosis effect in perimenopausal women as well (Liu et al. 2020). It is also found that vitamin K_2_ (Menaquinone) which is found in FSF like natto activates proteins such as osteocalcin (OC) and matrix-Gla protein (MGP), which are regulators of calcium distribution (Buchanan et al. 2016). Thus, studies suggest that vitamin K_2_ present in the traditional FSF such as natto has a role in bone metabolism. It may also contribute in maintaining BMD and in preventing osteoporosis (Jaghsi 2019).

#### Anti-diabetic property

Diabetes is a metabolic disease, which arises due to an increase in blood glucose level. In 2017, global incidence, prevalence, death, and disability-adjusted life-years (DALYs) associated with diabetes were 22.9 million, 476.0 million, 1.37 million, and 67.9 million, with a projection to 26.6 million, 570.9 million, 1.59 million, and 79.3 million in 2025, respectively (Lin et al. 2020). FSF such as meju have been reported to possess anti-diabetic property (Sanjukta et al. 2015). According to a cohort study conducted with 97,454 pregnant women, it is found that when there is an increase in isoflavone intake of about 10 mg/day, it resulted in 2% reduction of gestational diabetes. Similar results were observed with genistein and daidzein intake. Higher intake of miso soup and natto are also associated with a lower incidence of gestational diabetes (Dong et al. 2020).

Type 2 diabetes (T2D) is characterized by insulin deficiency and peripheral insulin resistance. Daidzein, an isoflavone, is predominantly present in the form of glucosides in FSF. It has been shown to have profound effects on the increased insulin resistance. Isoflavone leads to the breakdown of glycosidic linkage of these glycosides. 0.2 g daidzein/ kg body weight for 9 weeks led to the reduction in plasma glucose level in female mice while 0.2 g/ kg body weight for 6 weeks showed improvement of the glucose metabolism and regulation of the hepatic glucose (Das et al. 2018). It is reported that chungkookjang prepared with *B. amyloliquefaciens* may have anti-diabetic property that can potentially improve insulin sensitivity and insulin secretion capacity in a non-obese T2D animal model. This is due to the increased concentration of daidzein (Jeong et al. 2020).

The polymer which is produced outside the bacterial cell is known as poly-γ-polyglutamic acid (PGA). PGA production is one of the functional properties of *Bacillus* species and has many applications such as cryoprotectant, drug carrier, and biological adhesive. Its biodegradability property is used in various fields like medicine, food, and cosmetics (Chettri et al. 2016). A randomized crossover pilot study was conducted to investigate the suppression of the increased postprandial blood glucose level by the effect of γ-PGA present in natto. Blood samples were collected to analyze the blood glucose’s incremental area under the curve (IAUC), and it was found that the blood glucose’s IAUC of the high-γ-PGA natto meal was lower than the white rice and low-γ-PGA natto meal (all *p* < 0.05). The study observed suppression in the elevation of postprandial blood glucose level in the early phase after the simultaneous consumption of high-γ-PGA natto (Araki et al. 2020).

#### Cure for liver diseases

NAFLD (non-alcoholic fatty liver disease) is one of the most common comorbidities associated with metabolic syndrome (Kim et al. 2018). Studies have reported that FSF like doenjang exerts beneficial metabolic effects in diet-induced obese mice, which suppresses high-fat diet (HFD)-induced weight gain and reversed glucose intolerance and NAFLD-related metabolic parameters compared with control mice. This effect is due to the elevated active ingredients (flavonoids) by allowing beneficial fermenting microorganisms like *Bacillus* spp. to multiply (Kim et al. 2018). Several studies have reported that soybeans fermented by *Bacillus* spp. results in an increase in the amount of isoflavone (aglycone), and it shows high capability of flavonoid bioconversion. Long-term fermented soybean paste (LFSP), which is a *Bacillus* mixture, has shown to decrease HFD-induced weight gain and glucose intolerance. Mice treated with the *Bacillus* mixture shows significant glucose tolerance compared to untreated control mice (Kim et al. 2018).

#### Antioxidant property

Oxidation of biomolecules, cell death, and tissue damage take place in the human body due to the various biochemical processes. Antioxidants are molecules, which capture the free radicals and prevent oxidation (Moukette et al. 2015). It is reported that bioactive peptide purified from *B. subtilis* SHZ in FSF shows significant scavenging activity of superoxide (62%) and hydroxyl radicals (96%) at a concentration of 10 mg/ml (Sanjukta and Rai 2016). It has also been reported that yellow and black soybean fermented by *B. subtilis* MTCC 5480 possess higher antioxidant activity and it also aid in gastrointestinal digestion. Amino acids such as Trp, His, Phe, Ala, Tyr, Met, Gly, Leu, and Val have been observed to be the components of antioxidant peptides. Studies have shown that the proteolytic microorganisms like *Bacillus subtilis* present in FSF from Sikkim are reported to have enhanced antioxidant properties due to the increase in peptides as well as polyphenols. According to the findings, the fermentation of soybean increases radical (DPPH and superoxide) scavenging activity (3.1–24 folds) and total antioxidant activity in comparison with unfermented soybean. By-products resulting from processing soybean also show reasonably good antioxidant property. Hence, FSF can be used as an additive for functional foods and nutraceuticals to reduce oxidative stress (Sanjukta et al. 2015).

#### Enhance gut microbiota and immune system

In recent times, novel coronavirus (COVID-19) has been the cause for a huge health crisis across the world with increase in global morbidity and mortality rates. Appropriate intake of required nutrients could play a significant role by improving the immune response against the infection and by controlling the severity of the infection (Alkhatib 2020). A study from Japan has reported that FSF such as miso and natto etc have an essential impact in increasing the function of the immune system by maintaining the well-balanced gut microbiota. These foods have beneficial compounds like antioxidants, vitamins, minerals, folic acid, high protein, and probiotic bacteria which boost the immune system to fight against infections. Although there is evidence that FSF helps to promote health and improve the gut, there is no evidence regarding its effect directly on COVID-19 infection (Tashiro and Shaw 2020).

There is much evidence to support the statement that the traditional Chinese medicine shows beneficial effects in the treatment of SARS-CoV-2 patients. Yin qiao san is one of the traditional Chinese medicines (TCM) in which FSF is used as an ingredient along with various herbs (Fu et al. 2018; Ouassou et al. 2020). According to the theory developed by the TCM, the therapeutic effect of yin qiao san reduces toxicity, and this medicine is used to improve the function. It is also used to treat upper respiratory tract infection (Yang et al. 2020). It is believed that FSF containing *B. subtilis* (natto) has shown to enhance the growth of other microorganisms such as *Bifidobacterium*, *Lactobacillus*, *Escherichia coli*, and *Enterococcus* which improve the human gut microbiota (Dimidi et al. 2019).

#### Improving nutritional value with low cost and no side effects

Animal protein has been a major source for meeting the demand of protein rich foods, but due to its increased side effects, there is a strong incentive to use low-cost plant protein in the world economy. This demand has prompted the food industry to focus on the vegetable proteins in food formulations like FSF. Advances in processing technology have resulted in soy protein products that can perform numerous functions in the food industry while maintaining its excellent nutritional quality. As a result, soy protein products have widely been used and accepted as food ingredients to enhance the value of finished foods. In fact, they have become versatile that meat products, dairy, bakery, breakfast cereal, infant foods, and beverages may contain soy protein as a vital ingredient (Liu 2012).

### Health risk

Although many researchers have reported several beneficial effects of *Bacillus* spp. in the FSF, only a few researchers have reported some unfavorable effects of these *Bacillus* spp. on human health (Eom et al. 2015; Park et al. 2016)

#### Production of biogenic amines

Biogenic amines (BAs) are low molecular weight compounds formed by decarboxylation of amino acids or amination and transamination of aldehydes and ketones. Fermentation of soybean by the microbes may lead to the formation of BAs. These substances may lead to intoxication which causes symptoms in humans like nausea, respiratory distress, heart palpitation, and hypotension or hypertension. The formation of various vasoactive and putrefactive BAs (histamine, tyramine, β-phenylethylamine, tryptamine, putrescine and cadaverine) occurs commonly in the process of fermentation of the protein rich raw-materials like soybean, milk, meat, and fish (Lu et al. 2015; Tamang et al. 2016). Moreover, in the presence of nitrites, the compound biogenic polyamines (spermidine and spermine) are known to form carcinogenic nitrosamines. However, due to the presence of human intestinal amine oxidases (monoamine oxidase, diamine oxidase and polyamine oxidase), the oral intake of biogenic amines often does not cause any adverse effect. But symptoms may occur when the amine-metabolizing capacity is over-saturated, which takes place when metabolic activity is weakened by the inhibitors taken as a treatment for depression (monoamine oxidase inhibitors, phenelzine). The toxic dosage of biogenic amines in food is found to be 100–800 mg/kg of tyramine, 30 mg/kg of β-phenylethylamine, and 100 mg/kg of histamine. It is concluded that the levels of biogenic amines found in FSF may cause an adverse effect in sensitive consumers depending on the intake of amount and frequency of these products (Özogul and Özogul 2019).

*Bacillus* species (particularly *Bacillus subtilis*) are capable of decarboxylation of amino acids, which results in the production of BAs. A literature review has shown that *Bacillus subtilis* especially which ferments soybean contains ODC (ornithine decarboxylase) and LDC (lysine decarboxylase) proteins, which are responsible for putrescine and cadaverine production. In order to reduce the risk of intoxication from BAs, patients with depression, who are undergoing treatment with monoamine oxidase inhibitors (MAOIs), need to avoid ingesting fermented foods (Mah 2015).

#### Contamination by food pathogens

Food pathogens like *B. cereus* and *B. thuringiensis* are found in a wide variety of FSF. Food poisoning due to these strains may result in diarrhea and vomiting. This may be due to a complex enterotoxin produced by these strains called cereulide. *B. cereus* found at a concentration of more than 10^5^ CFU/g in food products induces food poisoning. According to a study, the major enterotoxin genes are found among the enterotoxigenic *B. cereus* group and emetic *B. cereus* isolates from the FSF like doenjang (Park et al. 2016). A study was conducted to evaluate the risk of *Bacillus cereus* in packaged tofu (from retail market to home) by quantitative risk assessment. This *B. cereus* could not grow at a temperature below 9 °C but were able to grow over 11 *°*C. But this *B. cereus* did not grow or die when the tofu was stored at 10 °C. The consumption of tofu can lead to foodborne illness, at about 1.0 × 10^−4^ per person per day. The control of biofilm formation may reduce the risks caused by *B. cereus*. In retail markets, monitoring the temperature aids in mitigating the risks caused by these microbes (Kwon et al. 2019; Kwon et al. 2020).

#### Occurrence of late-onset anaphylaxis

In a clinical review, 7 patients with suspected hypersensitivity to FSF were made to undergo skin prick test and challenge test. ELISA and Ig-E immunoblotting techniques were used to detect the serum specific IgE antibodies against the FSF and the allergens of natto extract. After ingestion of natto, all patients experienced generalized urticaria, dyspnea, vomiting, loss of consciousness, collapse, and diarrhea. Based on the results of IgE and skin prick test, the patients showed allergic reaction towards natto, but they were resistant to unfermented soybean and to the *Bacillus subtilis* (natto). It has been suspected that the natto allergens are produced during the fermentation. The mechanism of late onset of allergy remains unclear. The PGA is produced by *Bacillus* natto during fermentation. Studies have shown that some drugs can bind to the PGA for long-term controlled drug release (Richard and Margaritis 2003); similarly, since the natto allergens are bound to the PGA, it could take a long time to achieve a certain concentration to show symptoms. Therefore, it has been hypothesized that the reason for the late-onset of anaphylaxis is due to the binding of allergens with PGA, which is produced by *Bacillus* natto (Inomata et al. 2007)*.*

### Food safety

An effective method for improving the food safety and the functionality of FSF is by using *Bacillus* strains with desirable properties as starters. These starter strains can inhibit the growth of pathogenic microorganisms with its ability to produce bioactive compounds and also confer desirable organoleptic properties on the FSF.

Interventions to reduce the BAs in FSF have been done by the following methods: the use of irradiation, the addition of nicotinic acid (0.15% and 0.20%) as a tyrosine decarboxylase inhibitor, and the use of *Bacillus* starter cultures (*B. subtilis* and *B. amyloliquefaciens* strains) (Mah et al. 2019). Yet, another study has reported that the use of food additives (catechins and grapefruit seed extract) at different steps of the manufacturing process and the storage of soybean paste fermentation can reduce the production of biogenic amines. The levels of putrescine (PUT) and cadaverine (CAD) are decreased with the addition of grapefruit seed extract (300 mg/kg) and catechins (3 g/kg) respectively (Lee et al. 2018b; Mah et al. 2019).

A literature review has confirmed that the selected *B. licheniformis* strains from cheonggukjang are able to reduce the count of *B. cereus* from 3 to 4 log CFU/g to below l log CFU/g after fermentation (artificial contamination). *B. licheniformis* strains are capable of producing low levels of biogenic amines and good quality cheonggukjang (Su-Yeon et al. 2017). Similarly, *B. amyloliquefaciens* RD7-7 which is isolated from rice doenjang confirms the inhibition of toxin-related gene expression in *B. cereus* (Eom and Choi 2016).

Mah et al. reviewed various methods used to reduce the formation of BAs in FSF. In cheonggukjang, chemical intervention is used to inhibit the formation of BAs, particularly tyramine. The formation of BAs is reduced to 70% and 83% with the use of 0.15% and 0.20% concentration of nicotinic acid respectively. In doenjang, physical intervention (γ-radiation of raw materials) is used to reduce the contents of some BAs by 20–30%. It is noteworthy that the use of physical intervention may lead to delayed and abnormal fermentation. This is due to the decrease in the number of dominant bacteria which aids in fermentation. It is important to identify and characterize the starter strains at molecular level in order to regulate the expression of the genes involved in the formation of BAs (Mah et al. 2019).

Bacteriophages are viruses that infect bacteria and these are widely present in FSF (natto and cheonggukjang). These foods are hence known as fermented soybean bacteriophages (FSB). The phages which are capable of infecting bacterial pathogens can be considered beneficial. The FSB specific to *B. cereus* have been reported to exhibit strong lytic activity, which may reduce *B. cereus* contamination. In terms of problems in food safety, FSB could potentially be used as a biocontrol agent against other food borne pathogenic bacteria present in FSF like *E. coli*, *Salmonella* spp., and *Staphylococcus* spp. (Chukeatirote et al. 2018).

The accumulation or formation of BAs and the contamination by food pathogens in FSF are the causes for major health risk in humans. Hence, the solution to improve the food safety and quality is by using a defined starter culture with the absence of decarboxylase activity along with its beneficial metabolic and functional properties (bio-protection and BA degradation properties).

## Conclusion

We are in the post-genomics and proteomics era of microbiology where numerous researches have been conducted on microorganisms. Researchers have exploited microorganisms for the fermentation of food, to generate antimicrobial agents from genetic engineering and to produce valuable nutritional products. Microorganisms have been opening up new possibilities in the food and pharmaceutical industries. *Bacillus* spp., which are the key microorganisms present in FSF, have shown a plethora of applications in industries, and these species are included in the Food and Drug Administration's GRAS (Generally Regarded as Safe) list.

Ethnic fermented soybean food is a cost-effective source of plant protein which is traditionally a principal diet of the people of Southeast Asian countries. The *Bacillus* species present in these FSF not only renders in the process of fermentation but also produces several beneficial compounds such as isoflavones, lipopeptides, protein hydrolysates, and enzymes. The bioactive compounds in these FSF exhibit ACE inhibitory, antioxidant, anti-tumor, anti-diabetic, antimicrobial, anti-hypertensive, anti-allergic, and immunoregulatory properties. Studies on the bioactive compounds in FSF which have beneficial properties could be applied in the preparation of functional food and health supplements. Although the FSF’s nutritive values are concealed by the anti-nutritive properties, there are certain health risks associated with it. The clinical evidences have proclaimed that there are several health risks associated with FSF when it is consumed in immoderation. The ruinous effect produced by the *Bacillus* in FSF has resulted in food intoxication. Thus, it is considered to be a toxic substance for humans. Other detrimental effects pertaining to these foods are contamination by food pathogens like *B. cereus* and *B. thuringiensis* and late-onset anaphylaxis. On that account, the demand for safer food has resulted in the advancement in research linked to the non-beneficial effects of FSF. The knowledge involved in BAs production and the possible means of reducing its contents has been discussed in this review with the other health risks such as food poisoning by food pathogens and MAOIs. Hence, food safety measures are to be taken to monitor the levels of intoxicants present in the FSF.

To conclude, as the *Bacillus* species present in FSF have proven to show myriad of health benefits, these foods could be a robust option for human consumption only when efficient interventions are practiced to sustain food safety and quality.

## Future perspective

In recent years, scientific research on FSF-derived bioactive compounds, such as isoflavones, lipopeptides, protein hydrolysates, and enzymes, have been displaying a broad scope of functions. Though several studies have shown the activities and effects of these bioactive compounds in vitro, it cannot be directly linked to its in vivo effect. This is because the metabolic functions in terms of degradation and modification of these compounds may vary in in vivo conditions. Further, studies are yet to be made on the mechanism and activity of bioactive molecules produced by *Bacillus* spp. present in FSF against pathogenic microorganisms in order to commercialize it as beneficial products. As *Bacillus* species which are isolated from FSF are believed to have effective probiotic activity, hence these microbes can be used as a promising alternative to antibiotics in the world of rising antibiotic resistance. Studies on improving the safety and quality of these bioactive compounds for immunocompromised patients are also yet to be performed. Researches on the bioactive compounds in FSF are proved to have beneficial properties which could be applied in the preparation of functional food and health supplements.

## Data Availability

Not applicable

## Abbreviations

FSF: Fermented soybean food
LAB: Lactic acid bacteria
PGA: Poly gamma glutamic acid
ACE: Angiotensin I-converting enzyme
CVDs: Cardiovascular diseases
NK: Nattokinase
TGF: Transforming growth factor
SPM: Spermine
SPD: Spermidine
TNFα: Tumor necrosis factor alpha
COX2: Cyclooxygenase - 2
iNOS: Nitric oxide synthase
IL-6: Interleukin - 6
CPT-1: Carnitine palmitoyl transferase - 1
SBM: Soybean meal
BMP 2: Bone morphogenetic protein 2
OPG: Osteoprotegerin
BMD: Bone mineral density
OC: Osteocalcin
MGP: Matrix-Gla protein
DALYs: Disability-adjusted life-years
T2D: Type 2 diabetes
IAUC: Incremental area under the curve
NAFLD: Non- alcoholic fatty liver disease
HFD: High-fat diet
LFSP: Long-term fermented soybean paste
TCM: Traditional Chinese medicines
BAs: Biogenic amines
ODC: Ornithine decarboxylase
LDC: Lysine decarboxylase
MAOIs: Monoamine oxidase inhibitors
PUT: Putrescine
CAD: Cadaverine
FSB: Fermented soybean bacteriophages
GRAS: Generally Regarded as Safe
